# Cannabinoid type 1 receptor inverse agonism attenuates dyslipidemia and atherosclerosis in APOE∗3-Leiden.CETP mice

**DOI:** 10.1016/j.jlr.2021.100070

**Published:** 2021-03-23

**Authors:** Robin van Eenige, Zhixiong Ying, Lauren Tambyrajah, Amanda C.M. Pronk, Niek Blomberg, Martin Giera, Yanan Wang, Tamer Coskun, Mario van der Stelt, Patrick C.N. Rensen, Sander Kooijman

**Affiliations:** 1 Department of Medicine, Division of Endocrinology, and Einthoven Laboratory for Experimental Vascular Medicine, Leiden University Medical Center, Leiden, The Netherlands; 2 Center for Proteomics & Metabolomics, Leiden University Medical Center, Leiden, The Netherlands; 3 Center for Immunological and Metabolic Diseases, MED-X institute, and Department of Endocrinology, the First Affiliated Hospital of Xi'an Jiaotong University, Xi'an, China; 4 Department of Diabetes/Endocrine, Lilly Research Laboratories, Lilly Corporate Center, Indianapolis, IN, USA; 5 Department of Molecular Physiology, Leiden Institute of Chemistry, Leiden University, Leiden, The Netherlands

**Keywords:** adipose tissue, atherosclerosis, bile acids and salts/metabolism, cannabinoid receptor type 1, cardiovascular disease, drug therapy, endocannabinoids, lipids, lipoproteins/metabolism, BA, bile acid, BAT, brown adipose tissue, CB1R, cannabinoid type 1 receptor, CETP, cholesteryl ester transfer protein, E3L.CETP, APOE∗3-Leiden.CETP, gWAT, gonadal WAT, iBAT, interscapular BAT, LDLr, LDL receptor, RCT, reverse cholesterol transport, sWAT, subcutaneous WAT, TC, total cholesterol, TG, triglyceride, TRL, triglyceride-rich lipoprotein, UCP1, uncoupling protein 1, WAT, white adipose tissue, ZT, Zeitgeber time

## Abstract

Pharmacological blockade of the cannabinoid type 1 receptor, a G protein-coupled receptor expressed in the central nervous system and various peripheral tissues, reverses diet-induced obesity and dyslipidemia through the reduction of food intake and altered nutrient partitioning. This strategy is being explored for a number of therapeutic applications; however, its potency for the treatment of atherosclerotic cardiovascular disease via improvements in lipid metabolism remains unclear. Therefore, here, we aimed to investigate whether inhibition of the endocannabinoid system can attenuate atherosclerosis development through improvement of dyslipidemia. Lean, dyslipidemic female APOE∗3-Leiden.CETP transgenic mice were fed a Western-type diet supplemented with or without the cannabinoid type 1 receptor inverse agonist rimonabant (20 mg·kg body weight^−1^ day^−1^) for up to 20 weeks. Plasma lipids and bile acids were determined, and atherosclerotic lesions were scored in the aortic valve region. Rimonabant lowered plasma levels of triglyceride (TG) (−56%) and non-HDL-C (−19%) and increased HDL-C (+57%). These effects were explained by decreased VLDL-TG production (−52%) and accelerated VLDL-TG turnover accompanied by pronounced browning of white adipose tissue. In addition, rimonabant attenuated reverse cholesterol transport (−30%), increased plasma bile acid levels (+160%), and increased hepatic cholesterol accumulation (+88%). Importantly, rimonabant markedly lowered atherosclerotic lesion size (−64%), which coincided with decreased lesion severity (28% vs. 56% severe lesions) and which strongly correlated with non-HDL-C exposure (*R*^2^ = 0.60). Taken together, inhibition of the endocannabinoid system potently reverses dyslipidemia and prevents atherogenesis, even in the absence of obesity.

The majority of CVD is caused by atherosclerosis driven by inflammatory processes and dyslipidemia (1), which is characterized by high plasma non-HDL-C, high plasma triglyceride (TG), and low plasma HDL-C levels. Therefore, reverting dyslipidemia, and especially reducing non-HDL-C, is of key importance for reducing atherosclerosis and therefore the prevention of atherosclerotic CVD (2).

A potential strategy to revert dyslipidemia is stimulation of adipose tissue thermogenesis. Brown adipose tissue (BAT) converts energy stored in their intracellular lipid droplets into heat in the presence of uncoupling protein 1 (UCP1), which uncouples the mitochondrial electron transport chain from ATP synthesis (3). In addition, white adipose tissue (WAT) may acquire a more brown-like phenotype, a process referred to as browning that yields so-called beige adipocytes scattered between white adipocytes (4). To replenish internal lipid stores, thermogenic adipocytes selectively take up FAs after LPL-mediated liberation from triglyceride-rich lipoproteins (TRLs), thereby accelerating the generation and hepatic clearance of cholesterol-enriched TRL remnants (5, 6). During lipolysis, TRL surface remnants are transferred to HDL, which increases HDL-C levels and reverse cholesterol transport (RCT) (7). Taken together, activation of BAT thermogenesis is capable of lowering plasma TG and non-HDL-C levels while increasing plasma HDL-C levels, thereby attenuating dyslipidemia and protecting against atherosclerosis (8).

The main physiological activator of BAT thermogenesis is cold exposure, which results in the release of norepinephrine from sympathetic nerve endings within the tissue. Norepinephrine binds to β-adrenergic receptors on brown adipocytes, thereby activating adenylyl cyclase leading to cAMP production, intracellular lipolysis, and ultimately thermogenesis. This cascade is inhibited by cannabinoid type 1 receptor (CB1R) signaling, which prevents cAMP production through inhibition of adenylyl cyclase activity (9, 10). β-adrenergic stimulation elicits the production of endocannabinoids, the endogenous ligands for the CB1R, by thermogenic adipose tissue, which may act as a negative feedback mechanism to dampen thermogenesis (11). Indeed, we and others have previously shown that pharmacological blockade of the CB1R in vivo increases thermogenic BAT activity (12) and consequently attenuates dyslipidemia and protects from diet-induced obesity in mice (12, 13, 14). Moreover, CB1R inverse agonism has been shown to prevent atherosclerosis in ApoE^−/−^ and LDL receptor (LDLr^−/−^) mice (15, 16). However, as these mice lack an intact ApoE-LDLr pathway, these models largely preclude studying the antiatherogenic effects in the context of improvements in lipoprotein metabolism (5).

Attempts have been made to improve cardiometabolic health by inverse CB1R agonism using rimonabant in humans (17, 18, 19) and the early Rimonabant in Obesity-Lipids trial showed that rimonabant improved atherogenic dyslipidemia (20). Unfortunately, production and sale were suspended after being on the market for 2 years because of risk of serious psychiatric side effects (21). Interest in the endocannabinoid system as a therapeutic target dropped but recently gained new attention after the development of peripherally restricted CB1R antagonists (22) and endocannabinoid synthesis inhibitors (10, 23, 24). Therefore, in the current study, we continued studying the cardiometabolic benefits of CB1R modulation and aimed to investigate whether CB1R inverse agonism attenuates atherosclerosis development through reversal of dyslipidemia using APOE∗3-Leiden.CETP (E3L.CETP) mice, a well-established mouse model for human-like atherosclerosis development with an intact ApoE-LDLr pathway.

## Materials and methods

### Animals and diet

Homozygous human cholesteryl ester transfer protein (CETP) transgenic mice were crossbred with hemizygous APOE∗3-Leiden mice to generate E3L.CETP mice as detailed earlier (25). About 10–14-week-old mice were fed a Western-type diet containing 16% fat (15% cocoa butter and 1% corn oil) and 0.10% (experiment 1) or 0.15% cholesterol (experiment 2 and 3) (Altromin C 1000 mod. #100184 and #100185, respectively) for 3 weeks. Following this run-in period, in each experiment, mice were divided into two groups that were balanced for age, body weight, body composition, plasma TG levels, and plasma total cholesterol (TC) levels. Subsequently, mice were fed the respective Western-type diet supplemented with or without 20 mg·kg body weight^−1^ day^−1^ (0.0167%, w·w^−1^) rimonabant for 4 weeks (n = 7 with rimonabant, n = 8 without rimonabant, experiment 1; n = 8 per group, experiment 3) or 20 weeks (n = 15 per group, experiment 2). In experiment 2, one control mouse, because of malocclusion, and two rimonabant-treated mice, because of progressive ulcerative dermatitis, were excluded from all analyses. Mice were housed in conventional cages with a 12:12 h light-dark cycle with ad libitum access to food and water. All mouse experiments were performed in accordance with the Institute for Laboratory Animal Research Guide for the Care and Use of Laboratory Animals and had received approval from the Ethical Review Board for Animal Experimentation of the Leiden University Medical Center, Leiden, the Netherlands.

### Body weight, body composition, and food intake

Every 4 weeks (experiment 1 and 2), the mice were weighed, and body composition was determined using an EchoMRI-100 (EchoMRI). Food intake was measured by weighing and divided by the number of mice in each cage (n = 1 per cage, experiment 1; n = 4 per cage, experiment 2) to express the data as food intake in gram per mouse per day.

### Plasma lipid levels

Every 4 weeks (experiment 1 and 2), mice were fasted for 4 h (9:00 AM to 1:00 PM clock time, corresponding to Zeitgeber time [ZT] 2–6), and subsequently, blood was drawn from the tail vein in capillaries coated with paraoxon to prevent ongoing lipolysis. Blood plasma obtained after centrifugation was used to determine plasma TG and TC levels using enzymatic kits (10166588130 and 11489232216, respectively; Roche Diagnostics). Plasma HDL-C levels were determined in the supernatant after precipitation of ApoB-containing lipoproteins from plasma by addition of 20% polyethylene glycol 6000 (Sigma-Aldrich) in 200 mM glycine buffer (pH 10). From the TC and HDL-C levels, the non-HDL-C levels were calculated.

### Plasma levels of adhesion molecules

In 4-h fasted plasma samples collected as described above from 20-week treated mice (experiment 2), levels of intercellular adhesion molecule-1 and vascular cell adhesion molecule-1 were determined by ELISA using the manufacturer's protocols (MIC100 and MVC00, respectively; R&D Systems).

### Indirect calorimetry

In the fourth week of short-term intervention (experiment 1), mice were single housed in calorimetric cages (Promethion Line; Sable Systems International) to measure O_2_ consumption and CO_2_ production. After 5 days acclimatization, O_2_ consumption and CO_2_ production were measured at 5-min intervals for 2 consecutive days, from which the average energy expenditure, respiratory exchange ratio, carbohydrate oxidation rate, and FA oxidation rate were calculated (5). In the fourth week of experiment 2, mice were single housed, and feces samples were collected for determining bile acid (BA) levels as described below.

### BAT and WAT histology

At the end of the 4-week intervention period (experiment 1), mice were euthanized by CO_2_ inhalation, collected organs were weighed, and interscapular BAT (iBAT), gonadal WAT (gWAT), and subcutaneous WAT (sWAT) samples were fixated in 4% paraformaldehyde, dehydrated, and embedded in paraffin. Cross-sections of 5 μm were deparaffinized and treated with 0.3% H_2_O_2_ in 40% CH_3_OH to quench endogenous peroxidases (30 min). Sections were immersed in 10 mM citrate buffer at pH 6.0 (10 min, 97°C), blocked with 5% normal BSA in PBS (30 min, room temperature), and incubated overnight at 4°C with rabbit polyclonal anti-UCP1 (U6382; Sigma-Aldrich; 1:2000 in 1% BSA). After incubation, sections were incubated with HRP-labeled α-rabbit secondary antibody (K4003; DAKO EnVision™; 30 min, room temperature), which were then visualized by NovaRED™ HRP substrate (Vector Labs; 7 min, room temperature). Sections were counterstained with Mayers Hematoxylin (1.09249; Merck; 1:4 in H_2_O, 45 s, room temperature). Expression of UCP1 was quantified in iBAT and sWAT using ImageJ software (National Institutes of Health; version 1.52a), and stained area was expressed as a relative percentage of total lean area. Also using this software, the average adipocyte size was determined in gWAT and sWAT. Because of technical problems n = 6 per group remained for histological analysis.

### RCT assay

After 2 weeks of treatment with and without rimonabant (experiment 3), in vivo RCT was determined using a procedure originally developed by Zhang *et al.* (26). Four days before isolating peritoneal macrophages, donor female APOE∗3-Leiden mice received an intraperitoneal injection with 1 ml 3% Brewer's thyoglycollate medium (B2551; Merck). To load the recruited peritoneal macrophages with cholesterol, these donor mice received an intraperitoneal injection with 100 μg acetylated LDL and 125 μCi [1,2–^3^H(N)]-cholesterol (NET139001MC; PerkinElmer) 1 h before isolation by peritoneal lavage with 10 ml warm PBS. Macrophages were washed and resuspended in RPMI-1640 (30-2001; ATCC), and recipient mice were intraperitoneally injected with 2 ×·10^5^ macrophages and single housed for 72 h during which feces was collected. Feces samples (30–50 mg) were dissolved overnight at 55°C in 1 ml Tissue Solubilizer (Amersham Biosciences, Roosendaal, the Netherlands) with 200 μl 30% H_2_O_2_ after which ^3^H activity was determined in a liquid scintillation counter.

### Hepatic VLDL-TG and VLDL-ApoB production rate

To quantify VLDL production in vivo, after 4 weeks of treatment (experiment 3), mice were fasted for 4 h (07:00 AM to 11:00 AM clock time, corresponding to ZT 0–4) and anesthesized by a mixture of acepromazin, midazolam, and fentanyl (6.3, 6.3, and 0.3 mg/kg, respectively, intraperitoneally, followed by 31.3, 31.3, and 1.6 μg, respectively, subcutaneously every 45 min). Mice were subsequently intravenously injected with 10 μCi Tran[^35^S] label (IS-103; Hartmann Analytic) to label newly synthesized ApoB. About 30 min later, mice were intravenously injected with 5 μl/g body weight 10% Triton WR-1339 (T0307; Sigma-Aldrich) in PBS to block VLDL-TG clearance. Just prior to, and 15, 30, 60, and 90 min after injection with Triton WR-1339, blood was drawn from the tail vein, and plasma TG was determined as described above. After 120 min, mice were exsanguinated via the retroorbital sinus, and VLDL was isolated from serum after density gradient ultracentrifugation at *d* < 1.006 g/ml (27) by aspiration, in which ^35^S activity was determined using a liquid scintillation counter, and TG and TC was determined as described above.

### Plasma decay and organ uptake of VLDL-like particles

At the end of the 20-week intervention period (experiment 2), mice were fasted for 4 h (9:00 AM to 1:00 PM clock time, corresponding to ZT 2–6), blood was drawn as described above, and subsequently, mice were intravenously injected with glycerol tri[^3^H]oleate and [^14^C]cholesteryl oleate double-labeled VLDL-like particles (1.0 mg TG in 200 μl PBS per mouse) as described earlier (28). Blood was drawn from the tail vein at 2, 5, 10, and 15 min after injection to determine the plasma decay of both radiolabels. Then, mice were euthanized by CO_2_ inhalation, heart puncture plasma was collected, and mice were perfused with ice-cold PBS to remove blood from organs. Collected organs were weighed, and pieces were dissolved overnight at 55°C in Tissue Solubilizer (Amersham Biosciences, Roosendaal, the Netherlands), and ^3^H and ^14^C activity were determined in a liquid scintillation counter. Part of the liver was snap-frozen in liquid nitrogen and stored at −80°C for determining hepatic lipid content and mRNA expression as described below. The hearts were fixated in 4% paraformaldehyde, dehydrated, and embedded in paraffin for atherosclerosis quantification, as also described below.

### Hepatic lipid content

Liver lipids were extracted according to a modified protocol of Bligh and Dyer (29). Briefly, liver samples obtained from experiment 2 (approximately 50 mg) were cut and homogenized in 10 μl CH_3_OH per mg tissue. Lipids were extracted by addition of 1,800 μl CH_3_OH:CHCl_3_ (1:3 v·v^−1^) to 45 μl homogenate, followed by vigorous vortexing and centrifugation (15 min; 20,000 *g*). The organic phase was dried under a gentle stream of nitrogen and dissolved in 100 μl 2% Triton X-100 in CHCl_3_ and after another drying step dissolved in 100 μl H_2_O. TG and TC levels were determined as described above and expressed as nmol·mg^−1^ protein, for which protein concentration was determined using the Pierce™ bicinchoninic acid assay kit (23225; ThermoFisher).

### Hepatic gene expression analysis

Total RNA was extracted from liver samples (approximately 30–50 mg) obtained from experiment 2 using TriPure RNA Isolation Reagent (Roche Diagnostics) according to the manufacturer's protocol. RNA concentration was determined by NanoDrop (Thermo Scientific), and 1 μg cDNA was synthesized for each sample using Moloney Murine Leukemia Virus Reverse Transcriptase (Promega) according to the manufacturer's protocol. Quantitative real-time PCR was performed using GoTaq® qPCR Master Mix (A6002; Promega) with a Bio-Rad CFX96 Touch™ Real-Time PCR Detection System. mRNA expression levels were normalized to *Rplp0* mRNA expression and expressed as fold change using the 2^−ΔΔCT^ method. A list of used primer sequences is presented in Table 1.

**Table 1:** Primer sequences for quantitative real-time PCR

Gene	Primers	
	Forward	Reverse
*Abcg5*	TGTCCTACAGCGTCAGCAACC	GGCCACTCTCGATGTACAAGG
*Acaca*	AACGTGCAATCCGATTTGTT	GAGCAGTTCTGGGAGTTTCG
*Acta2*	ACTACTGCCGAGCGTGAGAT	AGGTAGACAGCGAAGCCA
*Angptl3*	ACATGTGGCTGAGATTGCTGG	CCTTTGCTCTGTGATTCCATGTAG
*Angptl4*	GGAAAGAGGCTTCCCAAGAT	TCCCAGGACTGGTTGAAGTC
*Angptl8*	CACTGTACGGAGACTACAAGTGC	GTGGCTCTGCTTATCAGCTCG
*Bsep*	CTGCCAAGGATGCTAATGCA	CGATGGCTACCCTTTGCTTCT
*Cidea*	CTCGGCTGTCTCAATGTCAA	CCGCATAGACCAGGAACTGT
*Cyp27a1*	TCTGGCTACCTGCACTTCCT	CTGGATCTCTGGGCTCTTTG
*Cyp7a1*	CAGGGAGATGCTCTGTGTTCA	AGGCATACATCCCTTCCGTGA
*Cyp7b1*	CAGCTATGTTCTGGGCAATG	TCGGATGATGCTGGAGTATG
*Cyp8b1*	GGACAGCCTATCCTTGGTGA	CGGAACTTCCTGAACAGCTC
*Fasn*	GCGCTCCTCGCTTGTCGTCT	TAGAGCCCAGCCTTCCATCTCCTG
*Hmgcr*	CCGGCAACAACAAGATCTGTG	ATGTACAGGATGGCGATGCA
*Il1b*	GCAACTGTTCCTGAACTCAACT	ATCTTTTGGGGTCCGTCAACT
*Il6*	ACCACGGCCTTCCCTACTTC	CTCATTTCCACGATTTCCCAG
*Ldlr*	GCATCAGCTTGGACAAGGTGT	GGGAACAGCCACCATTGTTG
*Ppargc1a*	TGCTAGCGGTTCTCACAGAG	AGTGCTAAGACCGCTGCATT
*Prdm16*	ACTTTGGATGGGAGCAGATG	CTCCAGGCTCGATGTCCTTA
*Rplp0*	GGACCCGAGAAGACCTCCTT	GCACATCACTCAGAATTTCAATGG
*Scd1*	GCGATACACTCTGGTGCTCA	CCCAGGGAAACCAGGATATT
*Tgfb1*	TGCGCTTGCAGAGATTAAAA	CTGCCGTACAACTCCAGTGA
*Tnfa*	AGCCCACGTCGTAGCAAACCAC	TCGGGGCAGCCTTGTCCCTT
*Ucp1*	TCAGGATTGGCCTCTACGAC	TGCATTCTGACCTTCACGAC

*Abcg5*, ATP-binding cassette subfamily G member 5; *Acaca*, acetyl-CoA carboxylase 1; *Bsep*, bile salt export pump; *Cidea*, cell death-inducing DFFA-like effector A; *Cyp27a1*, sterol 27-hydroxylase; *Cyp7a1*, cholesterol 7 alpha-hydroxylase; *Cyp7b1*, oxysterol and steroid 7-alpha-hydroxylase; *Cyp8b1*, sterol 12-alpha-hydroxylase; *Fasn*, fatty acid synthase; *Hmgcr*, HMG-CoA reductase; *Il1b*, interleukin 1 beta; *Il6*, interleukin 6; *Ldlr*, low density lipoprotein receptor; *Mttp*, microsomal triglyceride transfer protein; *Ppargc1a*, peroxisome proliferative-activated receptor gamma coactivator 1 alpha; *Prdm16*, PR/SET domain 16; *Rplp0*, ribosomal protein lateral stalk subunit P0; *Scd1*, stearoyl-CoA desaturase 1; *Tgfb1*, transforming growth factor beta 1; *Tnfa*, tumor necrosis factor alpha.

### Plasma and fecal BA levels

In heart puncture plasma and feces samples, BA levels were measured by LC/MS. Full details on chemicals, instrumentation, and method validation are provided in the supplemental data section. Briefly, 100 μl heart puncture plasma was added to 10 μl internal standard solution (500 ng/ml) and 290 μl CH_3_OH. Samples were vortexed (5 s) and stored at −20°C for 20 min. Then, samples were centrifuged (10 min; 18,213 *g*; 4°C), supernatant was dried under a gentle stream of nitrogen, and reconstituted by addition of 40 μl CH_3_OH followed by vortexing (5 s) and sonication (VWR USC 300 THD/HF; 1 min; maximum power; 22°C). About 60 μl H_2_O was added, and samples were vortexed (5 s) and centrifuged (10 min; 18,213 *g*; 4°C). About 80 μl supernatant was transferred to glass micro vial inserts, and samples were placed in the autosampler.

For feces samples, 75% CH_3_OH was added to approximately 40 mg sample to obtain a final concentration of 40 mg/ml. Samples were homogenized using a Next Advance Bullet Blender (BBx24B-CE; maximum speed; 10 min) after addition of three 3.2 mm stainless steel beads and a mixture of 0.9–2.0 mm stainless steel beads (approximately equal volume to feces sample). Subsequently, samples were vortexed (5 s) and 10 times diluted in 75% CH_3_OH. Samples were then vortexed (5 s), sonicated (1 min; maximum power, 22°C), vortexed (5 s), and centrifuged (5 min; 18,213 *g*; 20°C), after which 10 μl supernatant was transferred to glass micro vial inserts, and 10 μl internal standard solution (500 ng/ml) and 80 μl 50% CH_3_OH was added. Samples were then vortexed (5 s) and placed in the autosampler.

### Atherosclerosis quantification

Paraffin-embedded hearts were cross-sectioned (5 μm) throughout the aortic root area. Cross-sections were stained with hematoxylin-phloxine-saffron to determine lesion area as described previously (30). Lesions were categorized for lesion severity according to the guidelines of the American Heart Association adapted for mice (31) and categorized as mild lesions (type I–III) and severe lesions (type IV–V). Macrophages and smooth muscle cell actin were double stained using rat monoclonal anti-MAC-3 antibody (550292; BD Pharmingen) with Vector ImmPRESS® anti-rat (MP-7444; Vector Laboratories), and mouse monoclonal antiactin antibody (M0851; Dako) with DAKO EnVision™ anti-mouse, which were visualized with Vina Green (BRR807AH; Biocare) and 3,3′ diaminobenzidine (K3468; Dako), respectively. Collagen was stained with Sirius Red (1A280; Chroma). Lesion area and macrophage, smooth muscle cell, and collagen content were determined using ImageJ software (National Institutes of Health; version 1.52a).

### Statistical methods

Comparisons between groups were made by two-sided independent sample *t*-tests. Pearson product-moment correlation coefficients were determined to assess linear correlations between variables. *P* values less than 0.05 were considered statistically significant.

## Results

### CB1R inverse agonism lowers plasma TG and TC levels and stimulates WAT browning

**Fig. 1:**
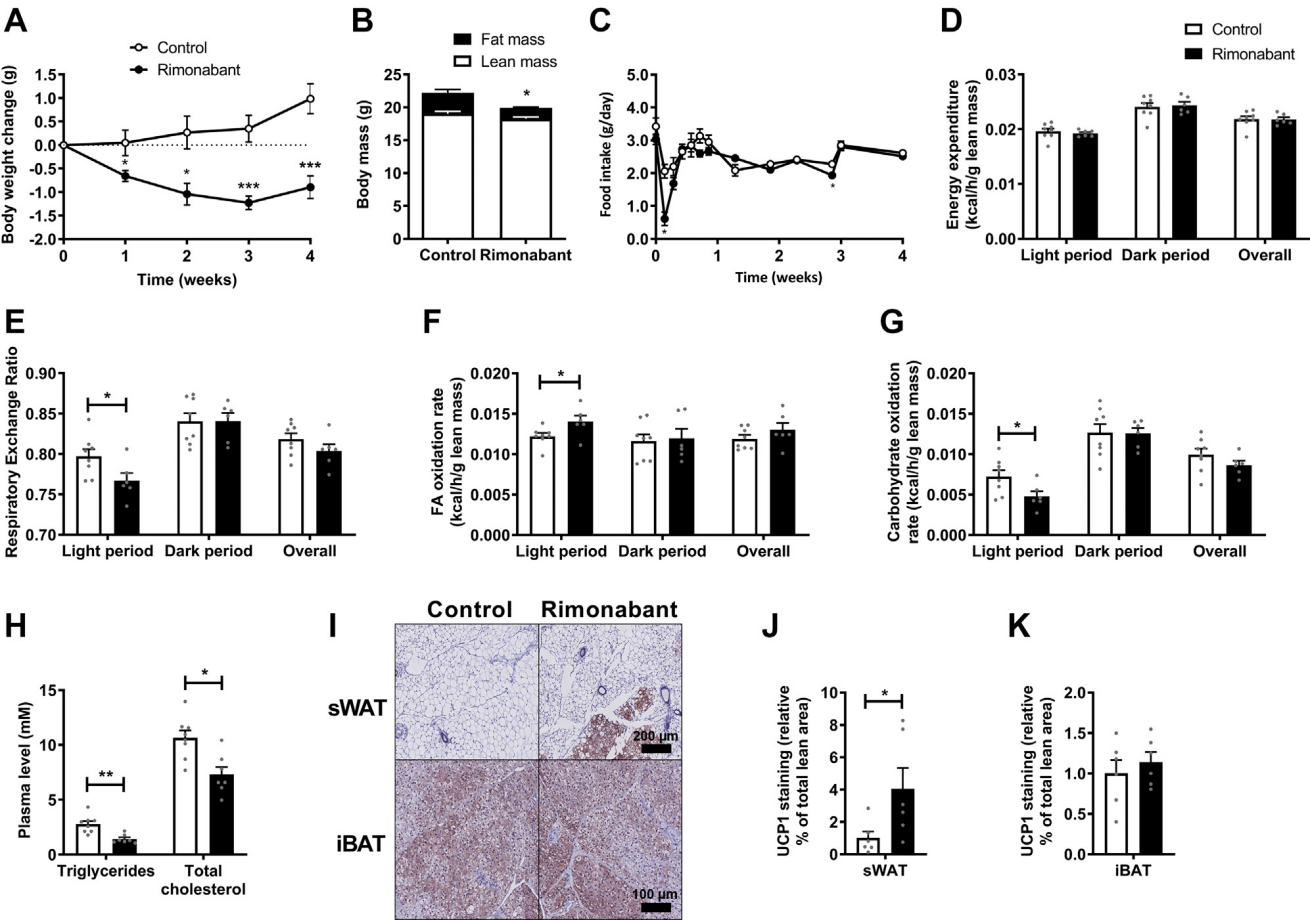
Rimonabant lowers plasma triglyceride and total cholesterol levels and stimulates white adipose tissue browning. Female APOE∗3-Leiden.CETP mice were fed a Western-type diet with or without supplementation of the cannabinoid type 1 receptor inverse agonist rimonabant for 4 weeks. A: Body weight was monitored weekly. B: Body composition was determined after 4 weeks of treatment. C: Food intake was measured throughout the entire intervention period. In the fourth week, mice were single housed in calorimetric cages to determine O_2_ consumption and CO_2_ production from which (D) energy expenditure, (E) respiratory exchange ratio, (F) FA oxidation rate, and (G) carbohydrate oxidation rate were calculated and expressed per gram lean mass. H: Plasma triglycerides and total cholesterol were determined in 4-h fasted blood plasma samples. Mice were sacrificed, and subcutaneous WAT (sWAT) and interscapular brown adipose tissue (iBAT) were collected, (I) stained, and (J, K) quantified for uncoupling protein 1 (UCP1). Data are presented as mean ± SEM and individual data points. ∗*P* < 0.05, ∗∗*P* < 0.01, and ∗∗∗*P* < 0.001.

In the first experiment, hyperlipidemic E3L.CETP mice were fed with or without supplementation of the CB1R inverse agonist rimonabant for 4 weeks to investigate the short-term effects on dyslipidemia, energy expenditure, and adipose tissue thermogenesis in an obesity-independent context. Rimonabant treatment decreased body weight throughout the intervention (Fig. 1A) by selectively lowering fat mass as compared with control (1.7 vs. 3.2 g after 4 weeks of treatment; Fig. 1B). Food intake was reduced during the first 2 days of rimonabant treatment but normalized thereafter (Fig. 1C). Total energy expenditure as measured by indirect calorimetry did not differ between rimonabant and control (Fig. 1D, supplemental Fig. S1A), but the respiratory exchange ratio was lower during the light phase (Fig. 1E) in rimonabant-treated mice because of increased FA oxidation at the expense of glucose oxidation (Fig. 1F–G). Without correction for lean mass, the increase in FA oxidation did not reach statistical significance (supplemental Fig. S1B, C). After 4 weeks of treatment, rimonabant treatment decreased plasma TG (−49%) and TC levels (−31%) (Fig. 1H). Liver weight was slightly increased in rimonabant-treated mice (+14%), and in concordance with the lowered fat mass, rimonabant reduced gWAT weight (−58%) and sWAT weight (−43%) (supplemental Fig. S1D) and reduced the average sWAT adipocyte size (supplemental Fig. S1E). Whilst gene expression levels of *Ucp1* and the browning markers *Ppargc1a*, *Cidea*, and *Prdm16* were not altered (supplemental Fig. S1F), rimonabant increased the UCP1 positive area in sWAT (+4-fold; Fig. 1I, J). UCP1 in iBAT was not changed (Fig. 1I, K). Altogether these data show that in lean E3L.CETP mice, rimonabant attenuates dyslipidemia, induces a shift from glucose to FA oxidation, and stimulates WAT browning.

### CB1R inverse agonism improves dyslipidemia as related to increased VLDL turnover and reduced hepatic VLDL production

**Fig. 2:**
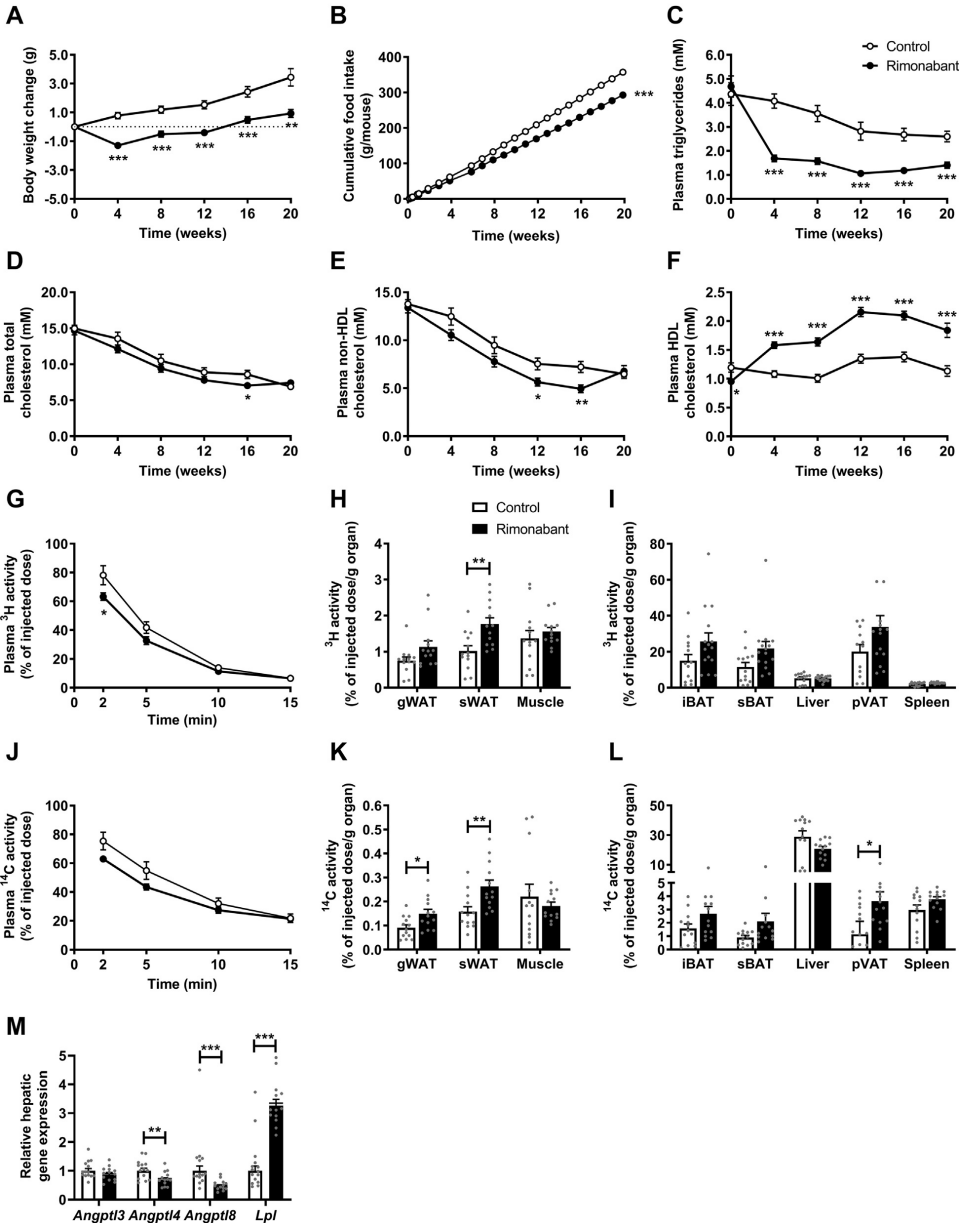
Rimonabant improves dyslipidemia as related to increased triglyceride (TG)-rich lipoprotein turnover. Female APOE∗3-Leiden.CETP mice were fed a Western-type diet with or without rimonabant for 20 weeks. A: Body weight, (B) food intake, as well as plasma levels of (C) triglycerides, (D) total cholesterol, (E) non-HDL-C, and (F) HDL-C were monitored throughout the intervention period. Plasma clearance and organ uptake of (G–I) TG-derived ^3^H-labeled FAs and (J–L) ^14^C-labeled cholesteryl ester from recombinant TG-rich lipoproteins were determined and expressed per gram gonadal WAT (gWAT) and subcutaneous WAT (sWAT), muscle, interscapular brown adipose tissue (iBAT), and subscapular brown adipose tissue (sBAT), liver, perivascular adipose tissue (pVAT), and spleen. Liver samples were collected to assess (M) relative mRNA expression levels of angiopoietin-like protein (*Angptl*) *3*, *4*, and *8* and of *Lpl*. Data are presented as mean ± SEM and individual data points. ∗*P* < 0.05, ∗∗*P* < 0.01, and ∗∗∗*P* < 0.001.

To investigate the long-term effects of CB1R inverse agonism on lipoprotein metabolism, we next treated E3L.CETP mice with or without rimonabant for a duration of 20 weeks. Throughout the intervention period, rimonabant slightly but consistently reduced body weight (Fig. 2A) and food intake (Fig. 2B). Consistent with the short-term study, rimonabant reduced plasma TG, which persisted throughout the entire intervention period (−62% to −46%; Fig. 2C). Rimonabant treatment modestly decreased plasma TC, which reached statistical significance only after 16 weeks of treatment (−18%; Fig. 2D). This was due to a lowering of non-HDL-C, reaching significance after 12 and 16 weeks of treatment (−25% and −32%, respectively; Fig. 2E), whereas HDL-C levels were increased throughout the entire intervention period (+46% to +62%; Fig. 2F). Plasma glucose after 20 weeks of treatment was not affected by rimonabant treatment in these lean mice (data not shown).

To elucidate whether increased lipolytic processing and plasma clearance of VLDL explains the reduction in plasma lipids within non-HDL, we next used VLDL-like particles containing glycerol tri[^3^H]oleate and [^14^C]cholesteryl oleate to investigate plasma clearance and organ uptake of TG-derived FA and VLDL core remnants, respectively. Compared with control, rimonabant treatment accelerated the clearance of glycerol tri[^3^H]oleate (Fig. 2G), which was accompanied by increased [^3^H]oleate uptake by sWAT (1.8% vs. 1.0%/g organ; Fig. 2H) and a nonsignificantly increased uptake by the various BAT depots (iBAT, 24% vs.15%/g organ; subscapular BAT, 21% versus 12%/g organ; perivascular adipose tissue, 32% vs. 20%/g organ; Fig. 2I). The clearance of [^14^C]cholesteryl oleate tended to be accelerated (Fig. 2J), with higher uptake by gWAT (0.15% vs. 0.09%), sWAT (0.26% vs. 0.16%), and perivascular adipose tissue (3.63% vs. 1.15%) (Fig. 2K, L). When data are expressed per whole organ, rimonabant increased both the uptake of [^3^H]oleate and [^14^C]cholesteryl oleate in subscapular BAT (0.59% vs. 0.34% and 0.06% vs. 0.03%, respectively; supplemental Fig. S2E, F). Consistent with increased lipolytic processing of plasma TG, rimonabant decreased hepatic gene expression of angiopoietin-like protein (*Angptl*) *4* (−31%) and *Angptl8* (−53%), known for their role as modulators of LPL activity, without affecting *Angptl3* expression, and increased *Lpl* expression 3-fold (Fig. 2M). Similar to the short-term study, rimonabant increased liver weight as compared with control (+22%) and lowered gWAT weight (−54%) as well as sWAT weight (−34%) (supplemental Fig. S2G).

**Fig. 3:**
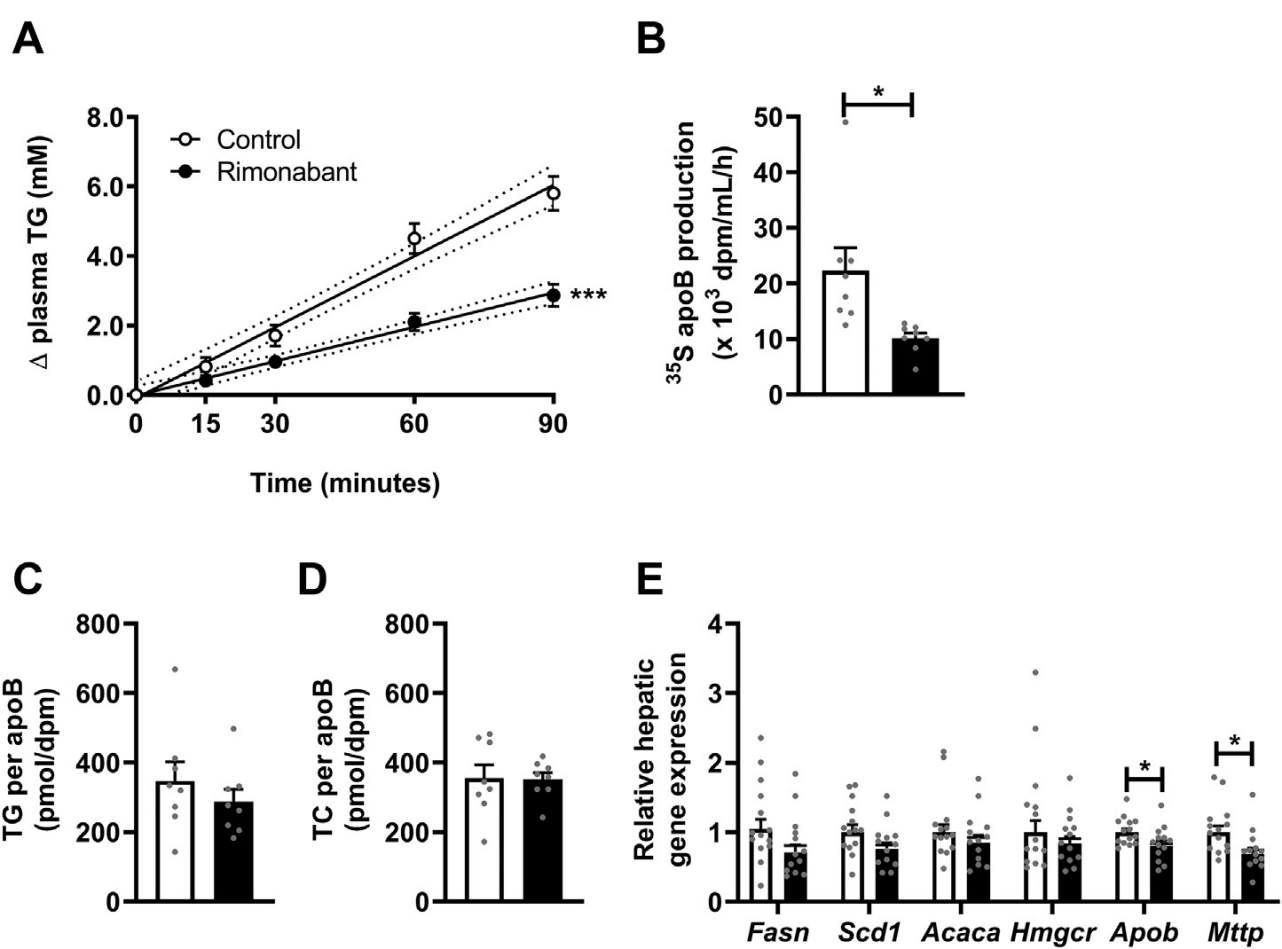
Rimonabant lowers hepatic VLDL-triglyceride (TG) and VLDL-ApoB production. Female APOE∗3-Leiden.CETP mice were fed a Western-type diet with or without rimonabant for 4 weeks. Mice received intravenous injection with Tran[^35^S] label followed by Triton WR1339, and TG levels were determined in plasma samples drawn at the indicated time points, which were (A) plotted as the increase in TG to baseline, from which VLDL-TG production rate was determined by linear regression. By measuring ^35^S in isolated VLDL (B), ApoB production rate was determined. C, D: VLDL lipid content was assessed and expressed as a ratio of ApoB. In 20-week treated mice, liver samples were collected to assess (E) relative mRNA expression levels of lipogenic (*Fasn*, *Scd1*, and *Acaca*) and cholesterogenic (*Hmgcr*) genes, as well as genes involved in VLDL assembly (*Apob* and *Mttp*). Data are presented as mean ± SEM and individual data points. ∗*P* < 0.05 and ∗∗∗*P* < 0.001. *Acaca*, acetyl-CoA carboxylase 1; *Fasn*, fatty acid synthase; *Hmgcr*, HMG-CoA reductase; *Mttp*, microsomal triglyceride transfer protein; *Scd1*, stearoyl-CoA desaturase 1; TC, total cholesterol.

As plasma TG levels are determined by both VLDL-TG clearance and production, we next investigated the in vivo VLDL-TG production rate as well as VLDL-ApoB production in an additional group of E3L.CETP mice treated with or without rimonabant for a duration of 4 weeks. Rimonabant strongly decreased VLDL-TG production (−52%) as well as VLDL-ApoB production (−55%) (Fig. 3A, B), without affecting TG/ApoB and TC/ApoB ratio (Fig. 3C, D). These changes were neither accompanied by significant reductions in the hepatic expression of lipogenic genes, such as *Fasn*, *Scd1*, and *Acaca*, nor a reduction in expression of the cholesterol synthesis gene *Hmgcr* in the 20-week treated mice, but correspondingly, rimonabant reduced the hepatic expression of *Apob* (−20%) and *Mttp* (−31%), both involved in VLDL assembly (Fig. 3E).

From these data, we conclude that rimonabant improves dyslipidemia through a combination of decreased hepatic VLDL particle production and increased VLDL turnover as a result of increased lipolytic activity in adipose tissue.

### CB1R inverse agonism influences BA metabolism and hepatic cholesterol

We previously observed that activation of thermogenic tissues lowers TC by increasing the total delivery of cholesterol from peripheral tissues to the liver (5, 7) coupled to increased hepatic BA synthesis. On the long term, this leads to increased plasma BA levels with negative feedback in the liver on expression of genes involved in BA synthesis, resulting in hepatic cholesterol accumulation (5, 32). Therefore, we next assessed the effects of rimonabant on fecal and plasma BAs, as well as on hepatic lipids in the E3L.CETP mice that were treated with or without rimonabant for 20 weeks.

**Fig. 4:**
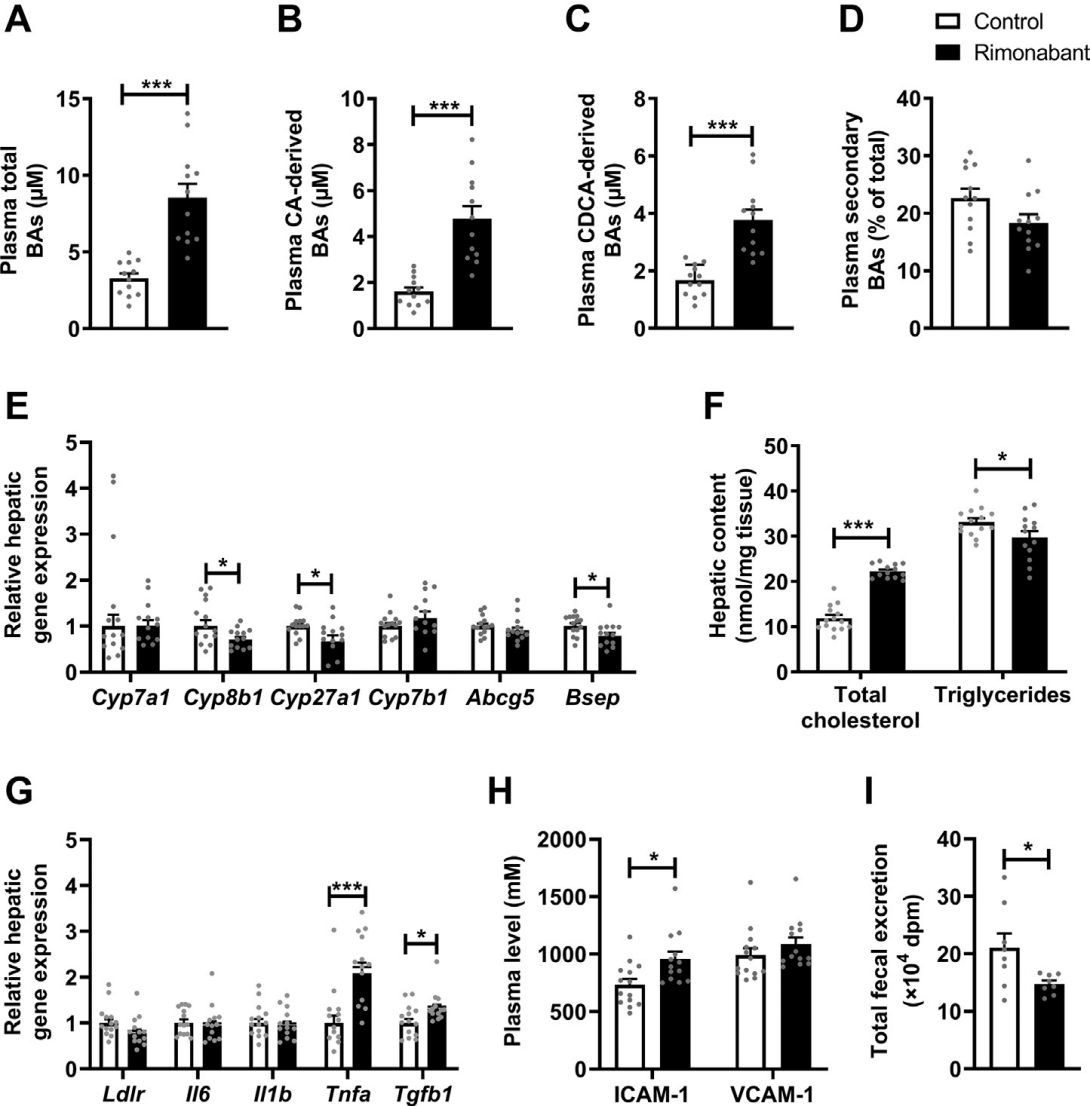
Rimonabant influences bile acid (BA) metabolism and hepatic cholesterol. Female APOE∗3-Leiden.CETP mice were fed a Western-type diet with or without rimonabant for 20 weeks. Mice were sacrificed, and heart puncture samples were collected to determine the plasma (A) total BAs, (B) cholic acid (CA)-derived BAs, and (C) chenodeoxycholic acid (CDCA)-derived BAs, as well as (D) secondary BAs as a percentage of total. Liver samples were collected to assess (E) relative mRNA expression levels of genes involved in the classical BA synthesis pathway (*Cyp7a1* and *Cyp8b1*), the alternative BA synthesis pathway (*Cyp27a1* and *Cyp7b1*), and the excretion of neutral sterols and cholesterol (*Abcg5* and *Bsep*), to assess (F) hepatic total cholesterol and TG content and to assess (G) expression of *Ldlr* and of various proinflammatory and profibrotic markers. H: In blood plasma collected after 20 weeks of treatment, plasma levels of intercellular adhesion molecule 1 (ICAM-1) and vascular cell adhesion protein 1 (VCAM-1) were determined. I: In an additional group of female APOE∗3-Leiden.CETP mice fed a Western-type diet with or without rimonabant for 2 weeks, mice received intraperitoneal injection with [^3^H]cholesterol-laden macrophages, and total fecal excretion of the radiolabel was determined during the following 72 h. Data are presented as mean ± SEM and individual data points. ∗*P* < 0.05 and ∗∗∗*P* < 0.001. *Abcg5*, ATP-binding cassette subfamily G member 5; *Bsep*, bile salt export pump; *Cyp27a1*, sterol 27-hydroxylase; *Cyp7a1*, cholesterol 7 alpha-hydroxylase; *Cyp7b1*, oxysterol and steroid 7-alpha-hydroxylase; *Cyp8b1*, sterol 12-alpha-hydroxylase; *Il6*, interleukin 6; *Il1b*, interleukin 1 beta; *Ldlr*, low density lipoprotein receptor; *Tgfb1*, transforming growth factor beta 1; *Tnfa*, tumor necrosis factor alpha.

Although rimonabant treatment did not alter total fecal BA excretion (supplemental Fig. S2A–D, supplemental Table S1), it markedly increased total BAs in plasma (+160%; Fig. 4A and supplemental Table S1), which was explained by increased levels of both cholic acid-derived BAs (+196%; Fig. 4B) and chenodeoxycholic acid-derived BAs (+126%; Fig. 4C). Consistently, rimonabant lowered the hepatic expression of *Cyp8b1* (−29%) and *Cyp27a1* (−33%), which are involved in the classical and alternative BA synthesis pathway, respectively (Fig. 4E). Rimonabant also reduced the hepatic expression of *Bsep*, involved in the excretion of BAs (−21%; Fig. 4E).

Probably because of impaired hepatic synthesis of BAs from cholesterol, rimonabant highly increased hepatic TC content (+88%), whereas slightly decreasing hepatic TG content (−10%) (Fig. 4F), which is consistent with a nonsignificant decrease of hepatic *Ldlr* expression (−22%, *P* = 0.06; Fig. 4G). Hepatic cholesterol accumulation was accompanied by increased hepatic gene expression of the proinflammatory cytokine *Tnfa* (+109%) and the profibrotic cytokine *Tgfb1* (+32%) (Fig. 4G) and by an increased plasma level of intercellular adhesion molecule-1 (+30%) but not vascular cell adhesion molecule-1 (Fig. 4H). To determine the impact of impaired BA synthesis, we next determined the rate of RCT by intraperitoneal injection of [^3^H]cholesterol-laden macrophages in E3L.CETP mice treated with or without rimonabant for a duration of 2 weeks and found that rimonabant decreased fecal ^3^H excretion (−30%; Fig. 4I). To conclude, in concordance with increased total delivery of cholesterol toward the liver combined with increased BAs in plasma and thus likely also in liver, prolonged treatment with rimonabant thus resulted in downregulation of hepatic BA synthesis, hepatic cholesterol accumulation, and reduced RCT.

### CB1R inverse agonism attenuates atherosclerotic plaque development

**Fig. 5:**
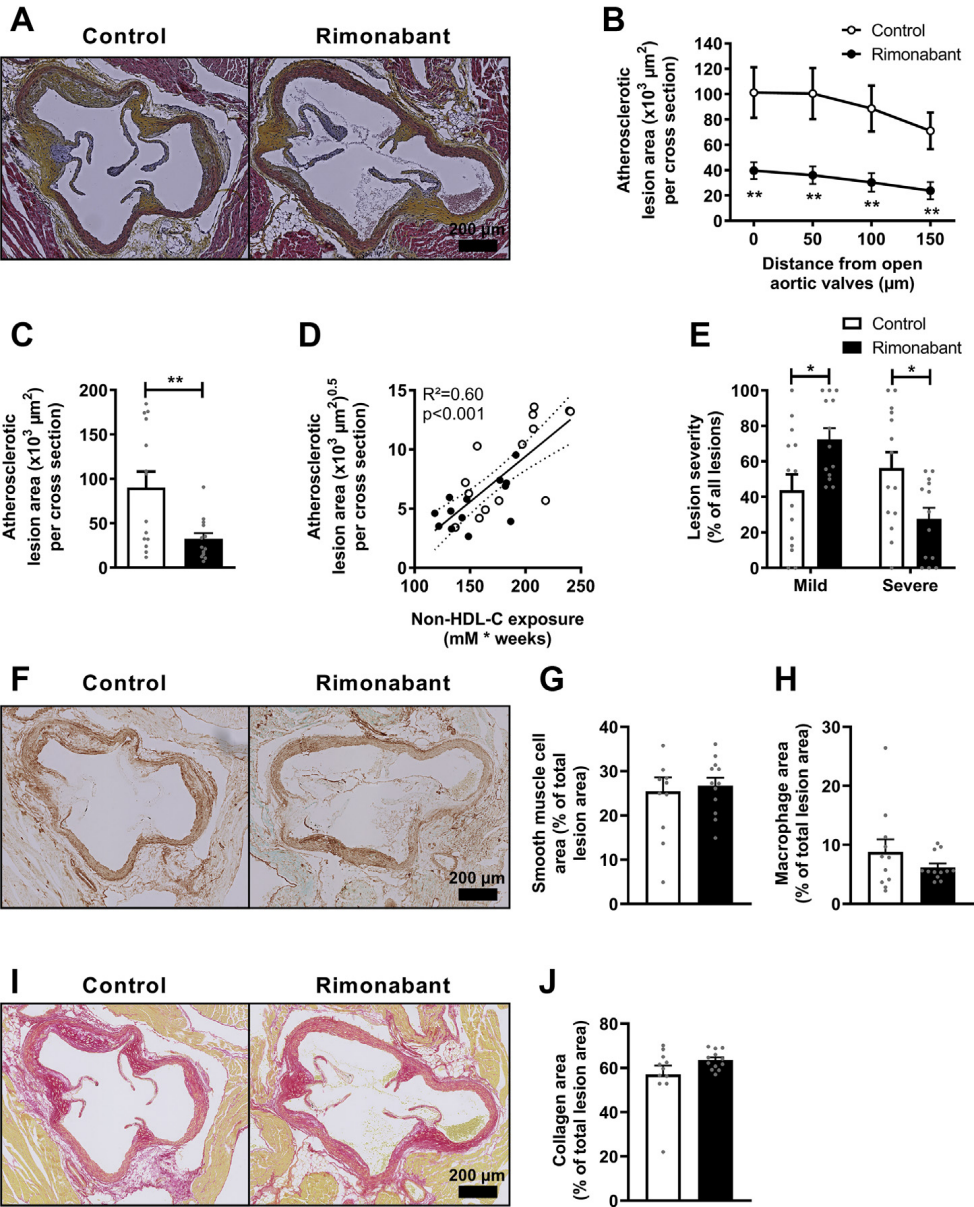
Rimonabant attenuates atherosclerotic plaque development. Female Western-type diet-fed APOE∗3-Leiden.CETP mice were fed with or without supplementation of the cannabinoid type 1 receptor inverse agonist rimonabant for 20 weeks. Mice were sacrificed, and hearts were collected. A: Cross-sections of the aortic root area were stained with hematoxylin-phloxine-saffron, and pictures of representative cross-sections are shown. B: Atherosclerotic lesion area was determined and is expressed as a function of distance from the appearance of open aortic valves, from which (C) the mean atherosclerotic lesion area was calculated. D: The square root of the mean atherosclerotic lesion area from C was plotted against the total non-HDL-C exposure. E: Lesions were categorized according to lesion severity and expressed as a percentage of total lesions. G: The smooth muscle cell (brown staining), (H) macrophage (green staining), and (J) collagen (red staining) area were measured within type III lesions, and (F, I) pictures of representative cross-sections are shown. Data are presented as mean ± SEM and individual data points. ∗*P* < 0.05 and ∗∗*P* < 0.01.

Next, to evaluate whether rimonabant attenuates atherogenesis in our mouse model, we quantified the atherosclerotic lesion area in the aortic root region of the hearts after 20 weeks of treatment. Atherosclerotic lesion area was markedly decreased by rimonabant (−64%; Fig. 5A–C) and showed a strong correlation with non-HDL-C exposure (control *R*^2^ = 0.54; β = 0.08, *P* < 0.01; rimonabant *R*^2^ = 0.41, β = 0.05, *P* < 0.05; Fig. 5D). Correspondingly, rimonabant decreased the overall lesion severity as compared with control, as indicated by a lower percentage of severe lesions (28% vs. 56%) and a higher percentage of mild lesions (72% vs. 44%) (Fig. 5E). Atherosclerotic lesion composition was determined in all lesions and within type III lesions only to correct for natural compositional discrepancies between the various lesion severity types. However, the relative content of collagen, smooth muscle cells, and macrophages was not altered by rimonabant either in all lesions (supplemental Fig. S2H–J) or in type III lesions only (Fig. 5F–J).

## Discussion

Since CB1R inverse agonism by rimonabant improves atherogenic dyslipidemia in mice (12, 13, 14) and humans (20), in the current study, we aimed to investigate whether rimonabant attenuates atherosclerosis development using hyperlipidemic E3L.CETP mice, a well-established mouse model for human-like lipoprotein metabolism because of an intact ApoE-LDLr pathway. We demonstrated that rimonabant reversed dyslipidemia by decreasing hepatic VLDL particle production and increasing VLDL turnover related to browning of WAT, together resulting in the prevention of atherosclerosis development.

We observed that CB1R inverse agonism potently lowers plasma TG levels, which was accompanied by accelerated plasma TG clearance as a result of increased TG-derived FA uptake by WAT, and a nonsignificantly increased FA uptake by BAT. These effects are consistent with enhanced thermogenic activity in adipose tissue and seem to be explained by pronounced browning of WAT related to a highly increased UCP-1 expression, rather than more active classical BAT. In a previous study in which we investigated the effect of rimonabant in the context of high-fat diet-induced obesity, we observed that rimonabant did increase the uptake of TG-derived FA by BAT (12). The seeming discrepancy between these studies is likely explained by the difference in dietary fat, which has recently been shown to acutely increase circulating endocannabinoid levels (33, 34). As higher endocannabinoid levels will reduce BAT activity by activation of CBRs, blocking the actions of endocannabinoids by rimonabant will potently restore BAT activity in the context of high-fat diet feeding. Indeed, the baseline FA uptake by BAT of E3L.CETP mice fed a high-fat diet (2%–5%/g organ) (12) was much lower than observed in the present study (10%–15%/g organ). This may also be relevant for humans as obese individuals have increased endocannabinoid levels when compared with lean individuals (35). Rimonabant not only increased VLDL turnover but also robustly decreased the production of VLDL-TG. Since this effect was paralleled by a similar decrease in VLDL-ApoB production, and each VLDL contains a single ApoB molecule, rimonabant apparently decreases VLDL particle production rather than lipidation of ApoB. Altogether in lean, Western-type diet-fed mice as used in the current study, CB1R inverse agonism decreases plasma TG levels and strongly attenuates atherosclerotic plaque development.

Besides lowering plasma TG, rimonabant decreased plasma non-HDL-C, increased plasma BAs, and increased hepatic cholesterol. These effects are consistent with previous reports that β3-adrenergic receptor agonism stimulates VLDL lipolysis accompanied by an enhanced flux of cholesterol-enriched VLDL remnants to the liver, which is efficiently coupled to higher production of BAs (32, 36). This results in higher fecal sterol output on the short term (36) but decreases fecal output and increases plasma BA levels on the long term (32). While increased systemic BA levels may be beneficial in further inducing thermogenesis (37) and likely related to the reduction in VLDL synthesis (38), they also provide negative feedback on hepatic BA synthesis (39). Indeed, rimonabant decreased hepatic expression of genes involved in the classical and alternative BA synthesis pathways, similar as we previously observed after prolonged β3-adrenergic receptor agonism (32). In line with this, RCT was strongly attenuated in rimonabant-treated mice. Whether the accumulation of BAs in plasma is caused by enhanced intestinal reuptake and/or by delayed BA clearance by the liver remains a topic for future studies. However, to prevent undesired hepatic cholesterol accumulation coupled to downregulation of hepatic BA synthesis, it would be interesting to investigate combined rimonabant treatment with BA sequestrants, which, as we previously demonstrated, largely increases BA excretion and effectively prevents hepatic cholesterol accumulation upon β3-adrenergic receptor agonism (32).

The close resemblance between the effects of CB1R inverse agonism and β3-adrenergic receptor agonism on hepatic cholesterol and BA metabolism may not be surprising as the CB1R and the β3-adrenergic receptor have closely coupled signaling pathways. To be specific, β3-adrenergic signaling promotes intracellular adenylyl cyclase activity, whereas CB1R signaling inhibits it (9). In fact, others have recently suggested that the endocannabinoid system acts as a negative feedback mechanism to dampen β-adrenergic signaling in the adipose tissues (11). It would thus be interesting to investigate whether β3-adrenergic agonism may synergize with CB1R inverse agonism to further attenuate dyslipidemia and atherosclerosis development.

Finally, rimonabant strongly reduced atherosclerotic plaque development, with a strong correlation between non-HDL-C exposure and atherosclerotic lesion size. This is similar to what we previously showed for, for example, β3-adrenergic agonism (5) and anacetrapib (40), and is fully consistent with the causal role of non-HDL-C in atherosclerosis progression (2). We thus interpret that rimonabant attenuates atherosclerosis by at least two ways, namely by decreasing the hepatic production of VLDL particles and accelerating the lipolytic processing of VLDL by thermogenic tissues, thereby lowering atherogenic non-HDL-C in the circulation and reducing its influx into the vessel wall. Our study complements the previous observations that rimonabant lowers atherosclerotic plaque development in ApoE^−/−^ and LDLr^−/−^ mice (15, 16). Since those mice lack an intact ApoE-LDLr clearance pathway for VLDL remnants, rimonabant did not reduce plasma cholesterol in those studies. Rather, the reduction in atherosclerosis was explained by enhanced RCT and a lowering of inflammation, respectively. This is in sharp contrast to our study, where we observed a marked reduction in RCT and a rather increased inflammatory status, both of which were possibly explained by the hepatic cholesterol accumulation. More mechanistic studies are needed to further confirm or refute a role for RCT and changes in inflammatory status in the atheroprotective effects of CB1R inverse agonism. Nevertheless, despite the discrepancies between the mode of action, our data at least strongly suggest a role for regulation of VLDL metabolism in the protecting effects of CB1R antagonism on atherosclerosis development. It is also interesting to note that a low dose of the cannabinoid derivative delta-9-tetrahydrocannabinol lowers atherogenesis in ApoE^−/−^ mice mediated via CB2R signaling on immune cells (41). More research is needed to determine the relative contribution of CB1R and CB2R signaling on atherosclerosis development; however, most data point toward an unfavorable effect of endocannabinoid signaling via CB1R.

Rimonabant will most likely not be reintroduced in the clinic (21). Adverse effects were caused by central modulation of the CB1R, and therefore, recent efforts were made to develop a new generation of CB1R antagonists that have limited to no brain penetrance. To even further improve safety, recent developments include so-called third-generation peripherally restricted dual-target CB1R agonists that may also have increased efficacy for certain metabolic diseases (22). Furthermore, other strategies to lower endocannabinoid tonus include inhibitors of the endocannabinoid synthesis enzymes (10, 23, 24). Together, these advancements hold promise for safely and effectively targeting the endocannabinoid system.

In conclusion, rimonabant decreases atherosclerosis development in lean hyperlipidemic E3L.CETP mice, as explained by decreased hepatic VLDL particle production and accelerated turnover of VLDL leading to a reduction in plasma non-HDL-C. Since E3L.CETP mice are a well-established model for human-like lipoprotein metabolism and atherosclerosis development, we anticipate that even in the absence of obesity, CB1R antagonism may be a promising strategy to combat CVD, probably through lowering lipids as well as inflammation. As the clinical development of rimonabant was discontinued owing to psychiatric side effects (21), peripherally restricted CB1R antagonists as well as inhibitors of the endocannabinoid synthesis enzymes may provide novel therapeutic handles to inhibit the endocannabinoid system in humans and may be used as a safe alternative for rimonabant in the future.

## Data availability

The datasets used and/or analyzed during the current study are available from the corresponding author on reasonable request.

## Supplemental data

This article contains supplemental data (42, 43, 44).

## Conflict of interest

T. C. is an employee and shareholder of Eli Lilly and Company. Eli Lilly and Company had no role in study design, data collection and analysis, decision to publish, or preparation of the article. All other authors declare that they have no conflicts of interest with the contents of this article.
