# Supplementary material for: Cannabinoid type 1 receptor inverse agonism attenuates dyslipidemia and atherosclerosis in APOE∗3-Leiden.CETP mice
Source: J Lipid Res. 2021 Mar 23;62:100070. doi: 10.1016/j.jlr.2021.100070 (PMC8082266; doi:10.1016/j.jlr.2021.100070)
Supplement: Supplemental data [file mmc1.pdf]

Supplemental information

Cannabinoid type 1 receptor inverse agonism attenuates dyslipidemia and atherosclerosis in APOE*3-Leiden.CETP mice

Robin van Eenige[a], Zhixiong Ying[a], Lauren Tambyrajah[a], Amanda C.M. Pronk[a], Niek Blomberg[b], Martin Giera[b], Yanan Wang[a,c], Tamer Coskun[d], Mario van der Stelt[e], Patrick C.N. Rensen[a,c], Sander Kooijman[a]

[a]Department of Medicine, Division of Endocrinology, and Einthoven Laboratory for Experimental Vascular Medicine, Leiden University Medical Center, Leiden, The Netherlands

[b]Center for Proteomics & Metabolomics, Leiden University Medical Center, Leiden, The Netherlands

[c]Center for Immunological and Metabolic Diseases, MED-X institute, and Department of Endcrinology, the First Affiliated Hospital of Xi'an Jiaotong University, Xi'an, China

[d]Department of Diabetes/Endocrine, Lilly Research Laboratories, Lilly Corporate Center, Indianapolis, IN, United States

[e]Department of Molecular Physiology, Leiden Institute of Chemistry, Leiden University, Leiden, The Netherlands

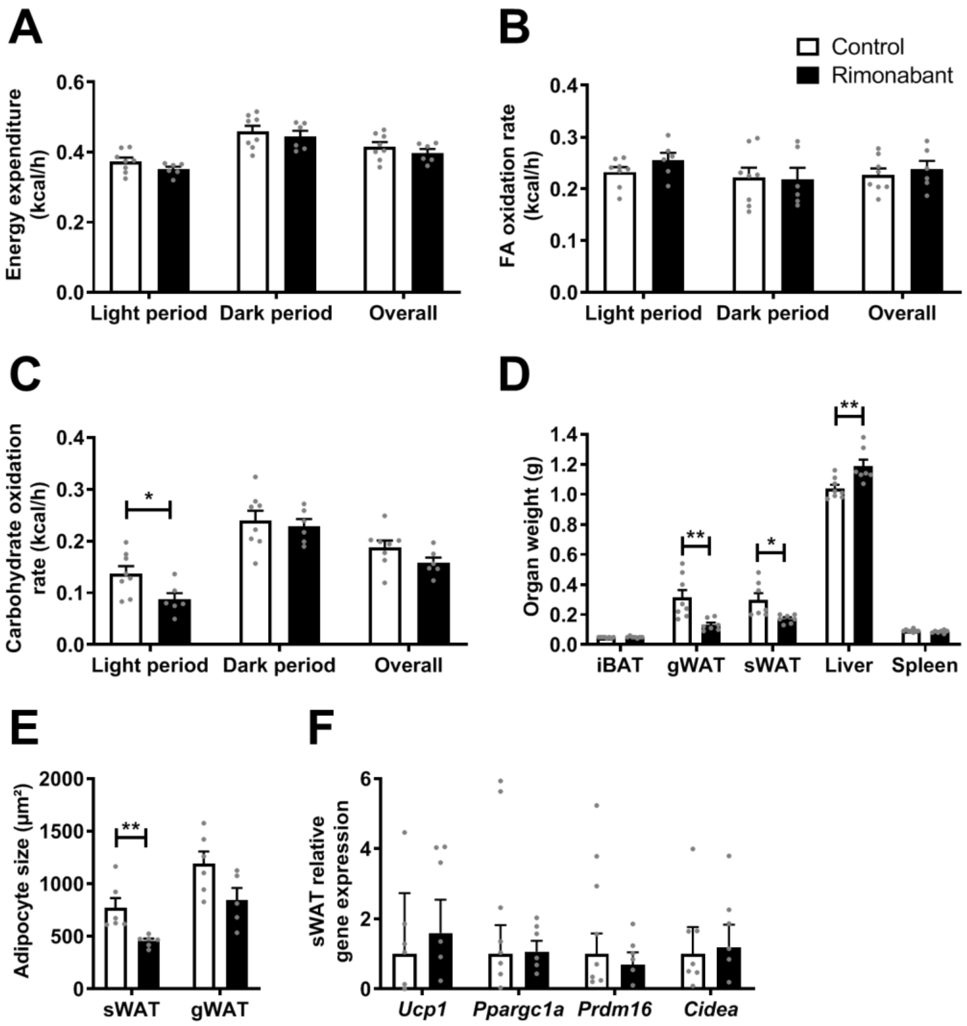

Supplementary Fig S1. Rimonabant increases liver weight and decreases adipocyte size. Female APOE*3-Leiden.CETP mice were fed a Western-type diet with or without supplementation of the cannabinoid type 1 receptor inverse agonist rimonabant for 4 weeks. In the fourth week, mice were single housed in calorimetric cages to determine O_2 consumption and CO_2 production from which **(A)** energy expenditure, **(B)** FA oxidation rate and **(C)** carbohydrate oxidation rate were calculated. Mice were sacrificed and **(D)** weights of the interscapular brown adipose tissue (iBAT), gonadal and subcutaneous white adipose tissue (gWAT; sWAT), liver and spleen were determined. **(E)** In sWAT and gWAT, the average adipocyte size was determined and (**F**) in sWAT relative mRNA expression levels of *Ucp1* and the browning markers *Ppargc1a, Prdm16* and *Cidea* were determined. Data are presented as mean ± SEM and individual data points. **P*<0.05, ***P*<0.01.

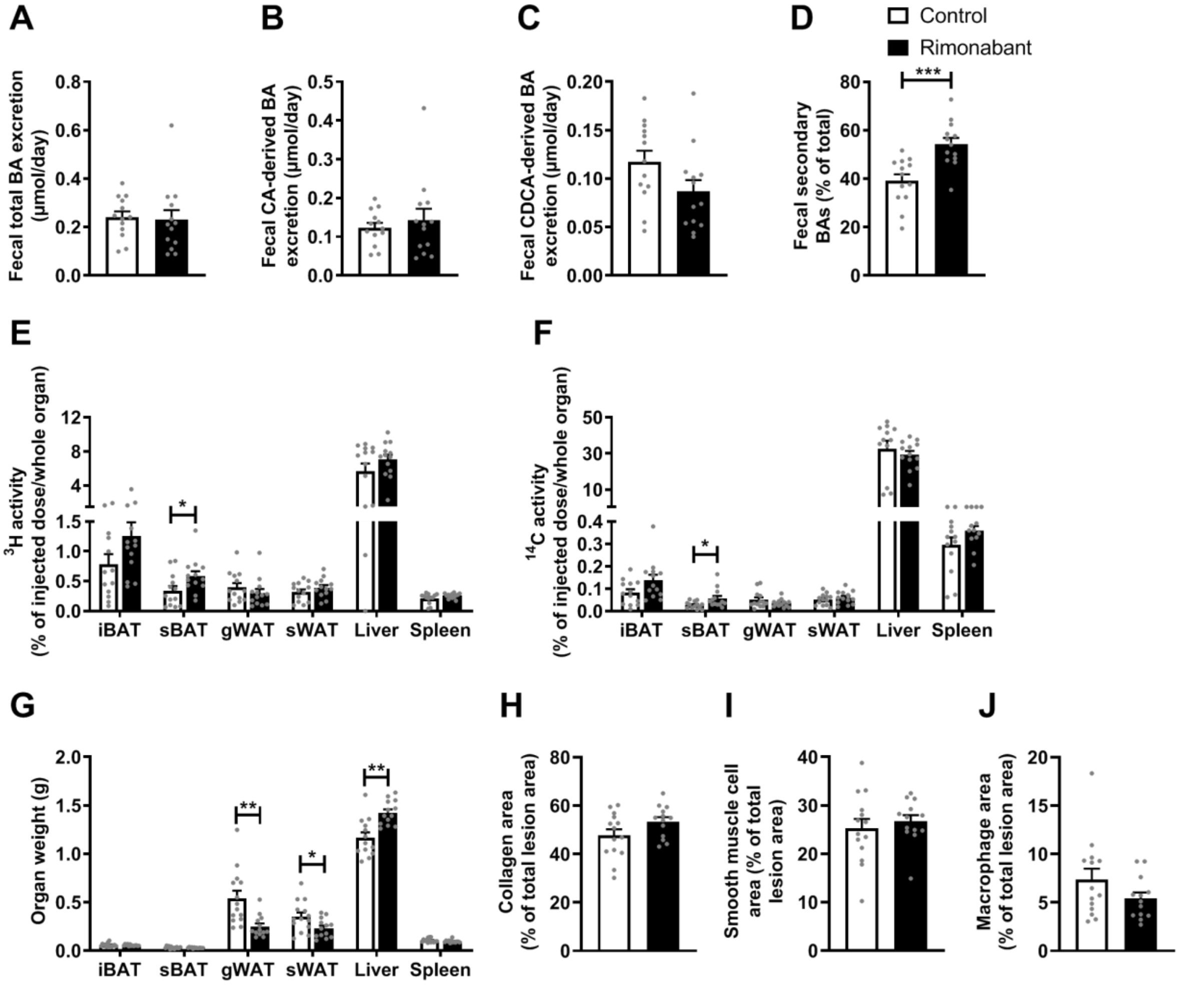

Supplementary Fig S2. Rimonabant does not alter fecal bile acid excretion. Female APOE*3-Leiden.CETP mice were fed a Western-type diet with or without rimonabant for 20 weeks. In the fourth week, mice were single housed and feces samples were collected to determine **(A)** total bile acid (BA) excretion, as well as **(B)** cholic acid (CA)-derived BA excretion, **(C)** chenodeoxycholic acid (CDCA)-derived BA excretion and **(D)** secondary BAs as a percentage of total. Mice were sacrificed, and organ uptake of **(E)** TG-derived ^{3}H-labelled FAs and **(F)** ^{14}C-labelled cholesteryl ester from recombinant TG-rich lipoproteins were determined and expressed per whole organ, for which **(G)** weights of the interscapular and subscapular brown adipose tissue (iBAT; sBAT), gonadal and subcutaneous white adipose tissue (gWAT; sWAT), liver and spleen were determined. Hearts were collected, cross-sections of the aortic root area were stained and the **(H)** collagen area, **(I)** smooth muscle cell area and **(J)** macrophage area were measured within atherosclerotic lesions. Data are presented as mean ± SEM and individual data points. *P<0.05, **P<0.01, ***P<0.001.

Supplementary Table S1. Rimonabant increases plasma bile acid levels, but does not alter fecal bile acid secretion.

Bile acid species	Plasma level (μM)		Fecal excretion (μmol/day)	
	Control	Rimonabant	Control	Rimonabant
αMCA	0.228 ± 0.029	0.435 ± 0.063**	n.d.	n.d.
βMCA	0.441 ± 0.063	0.834 ± 0.075***	0.087 ± 0.012	0.078 ± 0.008
CA	0.386 ± 0.044	0.636 ± 0.079*	0.028 ± 0.007	0.021 ± 0.011
UDCA	0.088 ± 0.008	0.125 ± 0.008**	0.002 ± 0.000	0.001 ± 0.000
HDCA	0.046 ± 0.004	0.101 ± 0.007***	n.d.	n.d.
DCA	0.546 ± 0.047	1.115 ± 0.105***	0.075 ± 0.011	0.118 ± 0.015*
CDCA	0.052 ± 0.005	0.073 ± 0.006*	0.002 ± 0.000	0.002 ± 0.000
G-CA	0.059 ± 0.002	0.076 ± 0.004***	n.d.	n.d.
G-DCA	0.039 ± 0.001	0.042 ± 0.001**	n.d.	n.d.
G-CDCA	0.039 ± 0.001	0.041 ± 0.001	n.d.	n.d.
T-αMCA	0.116 ± 0.028	0.202 ± 0.016*	n.d.	n.d.
T-βMCA	0.273 ± 0.046	0.892 ± 0.120***	0.010 ± 0.005	0.007 ± 0.003
T-CA	0.682 ± 0.171	3.030 ± 0.450***	0.003 ± 0.002	0.003 ± 0.002
T-DCA	0.225 ± 0.034	0.759 ± 0.118***	0.001 ± 0.000	0.001 ± 0.000
T-CDCA	0.054 ± 0.010	0.181 ± 0.018***	n.d.	n.d.
T-LCA	0.008 ± 0.001	0.009 ± 0.001	n.d.	n.d.

Female APOE*3-Leiden.CETP mice were fed a Western-type diet with or without rimonabant for 20 weeks. In the fourth week mice were single housed, feces was collected and at the end of the study mice were sacrificed and heart puncture blood samples were collected. Blood and feces samples were used to determine plasma levels and fecal excretion of alpha-muricholic acid (αMCA), beta-muricholic acid (βMCA), cholic acid (CA), ursodeoxycholic acid (UDCA), hyodeoxycholic acid (HDCA), deoxycholic acid (DCA), chenodeoxycholic acid (CDCA), glyco-cholic acid (G-CA), glyco-deoxycholic acid (G-DCA), glyco-chenodeoxycholic acid (G-CDCA), tauro-alpha-muricholic acid (T-αMCA), tauro-beta-muricholic acid (T-βMCA), tauro-cholic acid (T-CA), tauro-deoxycholic acid (T-DCA), tauro-chenodeoxycholic acid (T-CDCA) and tauro-lithocholic acid (T-LCA). Data are presented as mean ± SEM. *P<0.05, **P<0.01, ***P<0.001; n.d. is not detectable.

Supplementary Materials and Methods

Plasma and fecal bile acid levels

Chemicals and reagents. The following chemical standard materials were used: alpha-Muricholic acid (αMCA) and beta-muricholic acid (βMCA) were obtained from Steraloids Inc. (Newport, RI, USA); cholic acid (CA), ursodeoxycholic acid (UDCA), hyodeoxycholic acid (HDCA), deoxycholic acid (DCA), chenodeoxycholic acid (CDCA), glyco-cholic acid (G-CA), glyco-deoxycholic acid (G-DCA), glyco-chenodeoxycholic acid (G-CDCA), tauro-cholic acid (T-CA), tauro-deoxycholic acid (T-DCA), tauro-chenodeoxycholic acid (T-CDCA) and tauro-lithocholic acid (T-LCA) were obtained from Sigma-Aldrich (St. Louis, MO, USA). For each analyte an individual deuterium labelled internal standard was used: CA-d4, DCA-d4, G-CA-d5 and G-CDCA-d4 were obtained from Steraloids Inc. (Newport, RI, USA); UDCA-d4 was obtained from Cambridge Isotope Laboratories (Tewksbury, MA, USA); CDCA-d4, UDCA-d4, G-DCA-d5, T-CA-d5, T-CDCA-d5 and T-DCA-d5 were obtained from Toronto research chemicals (North York, ON, Canada).

Liquid chromatography–mass spectrometry (LC-MS)-grade methanol and water were obtained from Boom Chemicals (Meppel, NL). High-performance liquid chromatography (HPLC)-grade ammonium acetate and a 30-33% ammonium hydroxide solution were obtained from Sigma-Aldrich (St. Louis, MO, USA).

Instrumentation. A Shimadzu Nexera ultra-performance liquid chromatography (UPLC) system, consisting of two LC-30AD pumps, a SIL-30AC autosampler and a CTO-20AC column oven, coupled to a Sciex QTRAP 6500 with an electrospray ionization (ESI) source.

Liquid chromatography and mass spectrometry conditions. All measurements were performed using an Acquity UPLC BEH C18 column, 1.7 µm, 2.1 mm × 100 mm. This column was combined with an Acquity UPLC BEH C18 VanGuard pre-column (Waters, Milford, MA, USA).

Mobile phase A consisted of 100 mL CH_3OH and 900 mL water with 10 mM ammonium acetate adjusted to pH 8.5 using ammonium hydroxide. Mobile phase B consisted of 1 L CH_3OH and 10 mM ammonium acetate with addition of 120 µL ammonium hydroxide (42). The flow rate was set to 0.5 mL/min.

The chromatographic gradient started at 20% B and was held constant for 0.1 min, after which it was linearly increased to 55% B at 0.28 min, linearly increased to 73% B at 6.28 min, linearly increased to 100% B at 8.08 min and held constant for 2 min. The injection volume was 5 µL and the oven temperature was kept at 45°C.

For the detection of bile acids, the system was operated in the negative ESI mode. The following source settings were used: ion spray voltage, -4500 V; temperature, 600°C; curtain gas, 30; source gas 1, 30; source gas 2, 60; collision gas, medium; entrance potential, -10. The multiple reaction monitoring (MRM) transitions (42, 43) for all analytes and internal standards used, as well as their individual mass spectrometry parameters such as the declustering potential (DP), collision energy (CE) and the cell exit potential (CXP) are presented in Supplementary Table S2.

Supplementary Table S2. Multiple reaction monitoring transitions and mass spectrometry parameters for all analytes and internal standards used.

Analyte/Internal standard	Multiple reaction monitoring transition (Da)	Declustering potential (V)	Collision Energy (eV)	Cell exit potential (V)
**αMCA**	407.3 → 407.3	-180	-40	-20
**βMCA**	407.3 → 407.3	-180	-40	-20
**CA**	407.3 → 343.3	-180	-48	-30
**UDCA**	391.3 → 391.3	-180	-40	-20
**HDCA**	391.3 → 391.3	-180	-40	-20
**DCA**	391.3 → 345.3	-180	-48	-30
**CDCA**	391.3 → 391.3	-180	-40	-20
**LCA**	375.3 → 375.3	-180	-40	-20
**G-CA**	464.3 → 74.1	-150	-95	-33
**G-CDCA**	448.3 → 74.1	-150	-95	-33
**G-DCA**	448.3 → 74.1	-150	-95	-33
**T-βMCA**	514.3 → 80.1	-80	-130	-35
**T-CA**	514.3 → 80.1	-80	-130	-35
**T-DCA**	498.3 → 80.1	-80	-130	-35
**T-CDCA**	498.3 → 80.1	-80	-130	-35
**T-LCA**	482.3 → 80.1	-80	-130	-35
**CA-d4**	411.3 → 347.3	-180	-48	-30
**UDCA-d4**	395.3 → 395.3	-180	-40	-20
**CDCA-d4**	395.3 → 395.3	-180	-40	-20
**DCA-d4**	395.3 → 349.3	-180	-48	-30
**G-CA-d5**	468.3 → 74.1	-150	-95	-33
**G-CDCA-d4**	452.3 → 74.1	-150	-95	-33
**G-DCA-d5**	453.3 → 74.1	-150	-95	-33
**T-CA-d5**	519.3 → 80.1	-80	-130	-35
**T-CDCA-d5**	503.3 → 80.1	-80	-130	-35
**T-DCA-d5**	503.3 → 80.1	-80	-130	-35

Preparation of standard solutions and calibration curves.

Supplementary Table S3. Scheme to prepare the calibration curve.

**Standard**	**Final concentration (ng/mL)**	**Stock D (25 ng/mL; µL)**	**Stock C (250 ng/mL; µL)**	**Stock B (2.5 µg/mL; µL)**	**Stock A (25 µg/mL; µL)**	**Internal standard (500 ng/mL; µL)**	**CH_3OH (µL)**	**H_2O (µL)**
**1**	1	4	-	-	-	10	36	50
**2**	2	8	-	-	-	10	32	50
**3**	5	20	-	-	-	10	20	50
**4**	10	-	4	-	-	10	36	50
**5**	20	-	8	-	-	10	32	50
**6**	50	-	20	-	-	10	20	50
**7**	100	-	-	4	-	10	36	50
**8**	200	-	-	8	-	10	32	50
**9**	500	-	-	20	-	10	20	50
**10**	1000	-	-	-	4	10	36	50
**11**	2000	-	-	-	8	10	32	50
**12**	5000	-	-	-	20	10	20	50
**13**	10000	-	-	-	40	10	-	50

For the preparation of calibration curves, four stock solutions were made. Stock A (25 µg/mL), stock B (2.5 µg/mL), stock C (250 ng/mL) and stock D (25 ng/mL). The internal standard solution contained 500 ng/mL of each internal standard. All stocks were prepared with CH_3OH. A scheme for preparing the calibration curves can be found in Supplementary Table S3.

Method validation. Our method was validated on accuracy, matrix effects, recovery, repeatability and linearity.

Method accuracy was validated applying the standard addition method. Since bile acids are endogenous compounds, no biological matrix is bile acid free. Each bile acid was validated using 8-13 concentration points per calibration line for plasma and 5-6 concentration points per calibration line for feces, comparing fortified matrix with academic mixtures. All biological samples were corrected for endogenous bile acid levels.

Linearity in biological matrix was determined using 9 to 13 concentration points to construct a calibration line. Extraction repeatability was validated by adding bile acids to five different biological samples. These samples were then prepared, measured and the relative standard deviation (RSD; %) was determined.

Ion suppression/enhancement was determined according to Matuszewski *et al.* (44) comparing academic solutions with post-extraction spiked samples.

Extraction recovery was determined by comparing pre- and post-extraction samples, spiked with bile acids.

For all bile acids, the linearity in biological samples had $R^2>0.99$ with a range of 1 ng/mL to 500 ng/mL in feces and 1 ng/mL to 10,000 ng/mL in plasma. Intra-day repeatability ranged between 0.78% - 3.44% in plasma and 9.1% - 12.6% in feces. Accuracy, ion suppression and extraction recovery are presented in Supplementary Table S4.

Supplementary Table S4. Accuracy, ion suppression and extraction recovery.

	Accuracy (% ± 95% CI)		Ion suppression (% ± 95% CI)		Extraction recovery (% ± 95% CI)	
**Bile Acid**	**Plasma**	**Feces**	**Plasma**	**Feces**	**Plasma**	**Feces**
**αMCA**	91.4 ± 5.1	95.3 ± 16.3	80.7 ± 4.4	63.4 ± 24.9	78.3 ± 6.1	86.7 ± 6.3
**βMCA**	92.1 ± 4.8	98.0 ± 20.6	84.4 ± 5.5	81.5 ± 9.6	75.4 ± 6.3	81.4 ± 3.1
**CA**	96.9 ± 8.3	95.1 ± 3.5	84.2 ± 9.6	82.4 ± 2.4	76.4 ± 5.1	100.4 ± 6.0
**HDCA**	95.9 ± 4.4	94.4 ± 3.0	80.2 ± 5.3	-	78.5 ± 4.7	-
**UDCA**	94.4 ± 5.1	95.0 ± 2.3	77.3 ± 6.7	84.0 ± 8.0	79.4 ± 5.2	97.6 ± 1.8
**CDCA**	91.3 ± 5.1	94.5 ± 3.1	96.8 ± 16.7	80.5 ± 7.9	77.1 ± 5.0	101.1 ± 3.1
**DCA**	86.8 ± 5.0	97.5 ± 9.7	89.1 ± 7.4	75.4 ± 9.4	78.5 ± 4.1	102.1 ± 2.7
**G-CA**	89.9 ± 9.9	93.5 ±2.8	81.0 ± 7.8	79.0 ± 10.6	77.9 ± 1.3	94.1 ± 4.1
**G-CDCA**	94.3 ± 12.8	95.3 ± 2.7	87.8 ± 8.4	79.1 ± 3.8	75.7 ± 9.7	97.8 ± 4.3
**G-DCA**	97.2 ± 6.9	94.0 ± 2.9	102.0 ± 5.5	83.0 ± 17.3	75.6 ± 2.7	97.2 ± 5.1
**T-CA**	91.7 ± 8.0	93.4 ± 3.6	79.0 ± 9.4	80.9 ± 0.4	74.7 ± 5.4	99.1 ± 3.5
**T-CDCA**	77.0 ± 9.0	99.9 ± 2.9	72.1 ± 9.7	79.9 ± 3.6	73.6 ± 6.3	98.3 ± 3.1
**T-DCA**	77.6 ± 5.4	103.3 ± 3.0	114.9 ± 10.8	81.7 ± 6.4	73.0 ± 6.0	98.7 ± 4.5
